# Short-Term Clinical and Radiological Results of the Infinity Ankle Arthroplasty: A Prospective Study

**DOI:** 10.1177/19386400251330086

**Published:** 2025-04-21

**Authors:** Karen Sandvik Gyllensten, Ulf Sundin, Inger Storrønning, Ivar Ervik Husby, Erik Haavardsholm, Hilde Berner Hammer, Siri Lillegraven, Marianne Lund Eriksen, Mads Sundet

**Affiliations:** Center for Treatment of Rheumatic and Musculoskeletal Diseases (REMEDY), Diakonhjemmet Hospital, Oslo, Norway; Faculty of Medicine, University of Oslo, Oslo, Norway; Center for Treatment of Rheumatic and Musculoskeletal Diseases (REMEDY), Diakonhjemmet Hospital, Oslo, Norway; Faculty of Medicine, University of Oslo, Oslo, Norway; Center for Treatment of Rheumatic and Musculoskeletal Diseases (REMEDY), Diakonhjemmet Hospital, Oslo, Norway; Center for Treatment of Rheumatic and Musculoskeletal Diseases (REMEDY), Diakonhjemmet Hospital, Oslo, Norway; Center for Treatment of Rheumatic and Musculoskeletal Diseases (REMEDY), Diakonhjemmet Hospital, Oslo, Norway; Center for Treatment of Rheumatic and Musculoskeletal Diseases (REMEDY), Diakonhjemmet Hospital, Oslo, Norway; Faculty of Medicine, University of Oslo, Oslo, Norway; Center for Treatment of Rheumatic and Musculoskeletal Diseases (REMEDY), Diakonhjemmet Hospital, Oslo, Norway; Center for Treatment of Rheumatic and Musculoskeletal Diseases (REMEDY), Diakonhjemmet Hospital, Oslo, Norway; Center for Treatment of Rheumatic and Musculoskeletal Diseases (REMEDY), Diakonhjemmet Hospital, Oslo, Norway

**Keywords:** arthritis and joint disease, comorbid conditions, joint replacement, diagnostic & therapeutic techniques, reconstructive foot and ankle surgery, diagnostic & therapeutic techniques, surgical complications, general disorders, ankle injuries, heel—rearfoot—ankle

## Abstract

**Background:**

Ankle joint arthroplasty is increasingly used in primary or secondary end-stage osteoarthritis. Initial reports on the fourth-generation Infinity total ankle system showed a high failure rate due to component loosening. The objective of this study was to assess complications, reoperations, patient reported outcome measures (PROMs) and radiographic findings after 1 year.

**Methods:**

Data were prospectively collected in a local ankle surgery registry between January 2018 and March 2023. All cases of primary ankle arthroplasty using the Infinity system with a 1-year follow-up were included (106 ankles in 101 patients). Complications, revisions, and reoperations were described after 1 and 2 years, and the 1-year postoperative PROMs (AOFAS Ankle-Hindfoot score, MOxFQ, EQ5D, and EQ5D-VAS) were compared to preoperative values. One-year postoperative radiographs were assessed for signs of loosening.

**Results:**

The mean age was 65 years (SD 11.7), 55% were female, and the mean BMI was 27 kg/m^2^ (SD 4.2). Main indications for arthroplasty were chronic instability (27%), inflammatory joint disease (25%) and posttraumatic osteoarthritis (22%). After 1 year, there were 6 (5.5%) reoperations, of which 3 (2.7%) were prosthesis revisions, and 3 (2.7%) were outside the primary operative site. One (0.9%) reoperation was due to aseptic loosening of the tibia component. After 2 years, there was 1 additional reoperation with a tibial osteotomy and exchange of polyethylene insert. All PROMs improved significantly between the preoperative and the 1-year follow-up assessment, with mean change in AOFAS 35.9 (P < .01), MOxFQ 46.9 (P < .01), EQ5D 0.36 (P < .01), and EQ5D-VAS 13.2 (P < .01). There were findings of radiolucent lines and/or cysts on 1-year postoperative radiographs in 9 cases (9%).

**Conclusions:**

We found an acceptable rate of early aseptic loosening. All PROMs demonstrated a significant improvement from preoperative to 1-year postoperative assessment.

**Level of Evidence:**

Prospective cohort study, level II

“. . . outcomes of TAA in a relatively large number of cases in a real clinical setting at a medium sized center with no commercial affiliations”

## Introduction

Severe cases of ankle osteoarthritis have traditionally been treated by arthrodesis, providing pain relief at the cost of joint function. With advancing implant technology, total ankle arthroplasty (TAA) has become an increasingly appealing treatment option.^1^ TAA is well suited for the treatment of end-stage ankle osteoarthritis particularly in the presence of degeneration or previous fusion of adjacent mid- or hindfoot joints.^1^ In comparison to other major joint replacements such as in the hip or knee, long-term implant survival is relatively low in TAA. Data from the Norwegian Arthroplasty Register demonstrate a 10-year survival rate of 69.3% for TAA, while the 10-year survival rates for total hip and total knee replacements in Norway are approximately 95%.^2,3^

Over the last decades, there has been considerable development in design and fixation methods to improve TAA outcomes.^4^ The fourth-generation Infinity TAA system (Stryker, Kalamazoo, MI, USA) was introduced in 2014. The tibial component requires minimal bone resection, with a fixed bearing design.^5^ Early reports showed a high rate of early revisions due to symptomatic loosening of the tibial component.^6-8^ However, subsequent studies did not find an increased risk of loosening of the tibial component compared to previously used TAA systems.^9-13^

The objective of this study was to assess the rate of aseptic component loosening, type, and rate of other complications after 1 and 2 years, and to report clinical outcome measures and radiographic findings 1-year postoperatively.

## Methods

Our hospital is the main ankle arthroplasty center in Norway, performing more than 90% of the primary TAA and nearly all revision surgeries in the country. Data of all ankle arthroplasties has been systematically and prospectively recorded at predefined time points in our internal ankle replacement database. The Infinity ankle system was introduced at our hospital in October 2018, and has been the only implant used for primary ankle arthroplasties since then. The indication for ankle arthroplasty was primary or secondary end-stage osteoarthritis, causing disabling pain. The patients not eligible for ankle arthroplasty, such as patients with no preserved joint movement, young age, high demand for activity and unfavorable skin status, was offered an ankle arthrodesis. Contraindications to surgery were active infection, insufficient bone stock, large deformity, peripheral vascular disease or any medical conditions making the patient at high risk during surgery. The definitive choice of treatment was made through shared decision-making between patient and surgeon. In this study, we included all cases of primary TAA using the Infinity system (106 cases in 101 patients) with at least 1-year follow-up. Data were collected at the preoperative assessment visit, at the time of surgery, and at a 1-year postoperative clinical visit. Clinical outcome measures and range of motion were recorded by a physiotherapist, and an orthopaedic surgeon recorded details about the procedures, diagnoses, and comorbidities. Complications and reoperations were recorded continuously. Included patient data were collected from January 2018 until February 2024, at which time point 94.3% (n = 100) of the included cases had a follow-up time of at least 2 years, and the mean follow-up time of the patients was 45.8 (SD = 1.21) months.

Our internal ankle registry is classified as a quality database under Council of Norway research regulations, and a written informed consent is not mandatory. The patients were informed about the registry and the intended use of the data. The patients could refuse to participate or withdraw at any time. The database is approved by the hospital research board and data protection officer.

### Surgical Technique and Postoperative Care

All surgeries were performed by 1 of 2 orthopaedic foot and ankle surgeons with extensive experience with TAA (MLE and MS). The procedures were performed through an anterior approach to the ankle joint using standard instrumentation and technique with intraoperative fluoroscopy. Patients received concurrent surgery when indicated. All patients received antibiotic prophylaxis and thromboprophylaxis according to local procedure.

The patients were mobilized to crutches or other walking-aid before discharge, generally on the third postoperative day. The patients’ ankles were immobilized for 6 weeks; first in a cast for 3 weeks, and thereafter for 3 weeks in a Walker orthosis (Enovis Airselect Standard, DJO Nordic AB, Malmö, Sweden). Active and passive mobilization exercises were started 3 weeks after surgery. The patients were instructed to limit weight-bearing to 10 to 15 kg the first 3 weeks, and thereafter gradually increase the load to full weight-bearing after 6 weeks. During this period, patients were instructed to use crutches or other appropriate walking aids.

### Clinical Outcome Measures and Complications

We identified all complications and reoperations including revisions during the first 2 postoperative years. Complications were reported according to Glazebrook classification,^14^ and reoperations were reported according to the Canadian Orthopaedic Foot and Ankle Society (COFAS) reoperation coding system (CROCS) classification.^15^ American Orthopaedic Foot and Ankle Society –Ankle-Hindfoot score (AOFAS), Manchester Oxford foot and ankle questionnaire (MOxFQ), EQ5D-3L, and EQ5D-quality of life (EQ5D-VAS) were all completed preoperatively and after 1 year. We compared the preoperative and 1-year postoperative scores, and only cases with data present from both time points were included in these analyses (AOFAS/EQ5D n = 71, MOxFQ n = 67, EQ5D-VAS n = 68). The data were not complete, as the follow-up regime was designed for clinical purposes and not primarily for a scientific study, and a variety of reasons led to missing registrations. We assessed baseline BMI, age, sex, and primary diagnosis as predictors for 1-year change in each outcome measure.

### Radiographic Evaluation

Radiographs obtained 1 year postoperatively were evaluated by a specialist orthopaedic surgeon (KG), assessing the presence of periprosthetic radiolucent lines and cysts. A musculoskeletal radiologist was consulted (EH) in cases of uncertainty. A radiolucent line was defined as a hypodense zone in the bone-implant interface. A cyst was defined as a hypodense zone greater than 5 millimeter in diameter in proximity to the implant.^7^ The radiological department used a standardized protocol for the postoperative images, where the images are recorded orthogonal to the implant with the assistance of fluoroscopy. Of the 106 TAA cases, 95 had 1-year follow-up radiographs and were included in these analyses.

### Statistical Analyses

Baseline characteristics and diagnosis/surgical indication were described as means or percentages as appropriate. Complications, reoperations, and abnormal radiographic findings were described as number of cases and percentages by type or classification. For the outcome measure instruments, we compared preoperative and 1-year postoperative mean scores (delta, ∆) using paired sample t–tests. We used unadjusted linear regression to calculate if there were any statistically significant associations between ΔAOFAS or ΔMOxFQ and BMI, age, sex, or primary diagnosis. We used box plots to visualize the change form baseline in AOFAS and MOxFQ scores grouped by sex, age, BMI, and diagnosis. The statistical analyses were performed with STATA BE 17.0 (StataCorp LLC, College Station, TX, USA). The statistical analysis was made by the main authors (KG and US), and a statistician were consulted when needed. The article is written following the principles given in the STROBE-statement.^16^

## Results

### Study Population, Demographics, and Characteristics

106 ankles were operated in 101 patients, with a mean age at the time of TAA of 65.0 years (SD = 11.9) (Table 1). There was a slight predominance of women (55%), who were on average 6 years younger than the men at the time of primary surgery (62 vs 68 years *P* < .01). The mean BMI was 27 kg/m^2^ (SD 4.2). The main indications for surgery were osteoarthritis secondary to chronic instability, joint destruction due to inflammatory joint disease, and posttraumatic osteoarthritis. Concurrent surgical procedures were performed in 30 cases, and varied from simple Achilles lengthening (n = 23), removal of hardware (n = 2), filling cysts (n = 1), or other minor ligamentous procedures (n = 2), to more extensive surgeries such as calcaneal/subtalar/tarsometatarsal osteotomies (n = 4), and fixation of perioperative fracture (n = 1). Three patients received more than 1 concurrent procedure.

**Table 1.** Baseline Characteristics.

Patient characteristics	Number of cases = 106
**Age, years**	
* Overall*	65.0 (11.9)
* Male*	68.3 (10.4)
* Female*	62.4 (12.4)
Gender, female	58 (54.7%)
Body mass index, kg/m2	27.1 (4.2)
**Diagnosis**	
Chronic instability	28 (26.4%)
Inflammatory joint disease	26 (24.5%)
Posttraumatic osteoarthritis	24 (22.6%)
Primary osteoarthritis	10 (9.4%)
Hemochromatosis	7 (6.6%)
Other	6 (5.7%)
Hemophilia	3 (2.8%)
Infection-sequela	2 (1.9%)

Dichotomous variables presented as n (proportion), continuous variables as mean (SD).

### Reoperations and Complications

During the first year following the primary surgery, there were 6 reoperations, of which 3 were component revisions (Table 2, Table 3). Other complications were 1 intraoperative fracture that was fixated during the primary operation, and 4 patients who experienced altered sensation after 1 year, mainly on the plantar aspect of the foot. Of the 100 cases with 2 years follow-up time, there was 1 additional revision.

**Table 2.** Overview of Complications.

Grade	Complication	N (%)
High	Deep infection	1 (0.9 %)
	Aseptic loosening	1 (0.9 %)
	Implant failure	1 (0.9 %)
Medium	Technical error	0 (0.0 %)
	Subsidence	0 (0.0 %)
Low	Postoperative bone fracture	1 (0.9 %)
	Intraoperative bone fracture	1 (0.9 %)
	Wound healing problems	2 (1.8 %)
**Total complications**		**7 (6.6%)**

Complications classified according to the Glazebrook classification.^14^

**Table 3.** Complications Resulting in Reoperations.

Reoperation no	Etiology of arthritis	Preoperative coronal plane deformity	Postoperative coronal plane deformity	Time to reoperation (months)	Reason for reoperation (CROCS type)	Action
*Reoperations within first year*						
1	Inflammatory joint disease	24° varus	0	1	Deep infection (10)	Three stage revision to hindfoot nail with tantalum cage
2	Inflammatory joint disease	13° varus	1° varus	1	Superficial infection (3)	Wound debridement
3	Chronic instability	10° valgus	2° varus	3	Fracture and impaction of tibia component (9)	One stage revision to Inbone/Invision
4	Inflammatory joint disease	5° varus	5° varus	2	Skin necrosis (3)	Wound debridement, skin graft
5	Haemochromatosis	0	0	0	Fracture of medial malleolus (3)	Fixation of the fracture
6	Inflammatory joint disease	0	0	10	Aseptic loosening of the tibial component (9)	2 revisions, last to Inbone/Invision
*Reoperations during second year*						
7	Inflammatory joint disease	12° varus	5° varus	18	Malalignment (4/5)	Tibial osteotomy, polyethylene liner exchange

COFAS Reoperation Coding System (CROCS) classification^15^ 1. No reoperation 2: Isolated hardware removal 3: Reoperation outside initial operative site 4: Gutter or heterotopic ossification debridement 5: Polyethylene liner exchange 6: Debridement of osteolytic cyst 7: Deep infection requiring debridement 8: Revision arthrodesis 9: Revision of metal components do to implant failure 10: Infection requiring revision of metal components 11: Amputation above the level of ankle.

#### Reoperations within the first year

There was 1 case of early deep surgical site infection, which despite several attempts at soft-tissue debridement ultimately required ankle fusion by retrograde intramedullary nailing with a tantalum cage insert (Figure 1).

**Figure 1.**
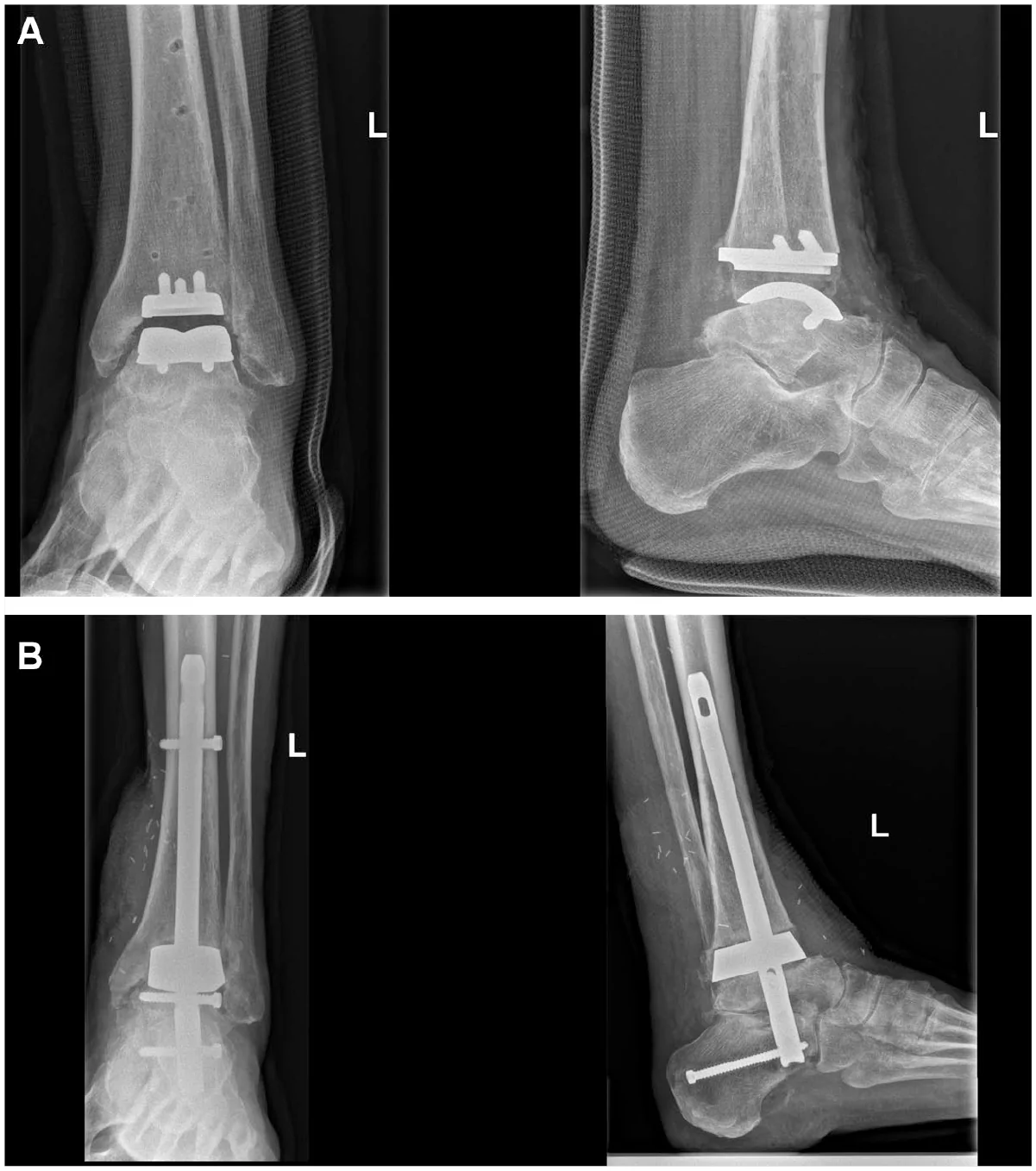
Revision due to deep infection: (A) postoperatively and (B) after revision to hindfoot fusion.

There was 1 case of aseptic loosening of the tibial component, resulting in 2 reoperations. At the first revision, the loose component was replaced by a cemented implant. However, this also showed signs of early loosening after a few months and was consequently revised using an Invision tibial component (Stryker, Kalamazoo, MI USA) and an Inbone talus component (Stryker, Kalamazoo, MI USA) (Figure 2).

**Figure 2.**
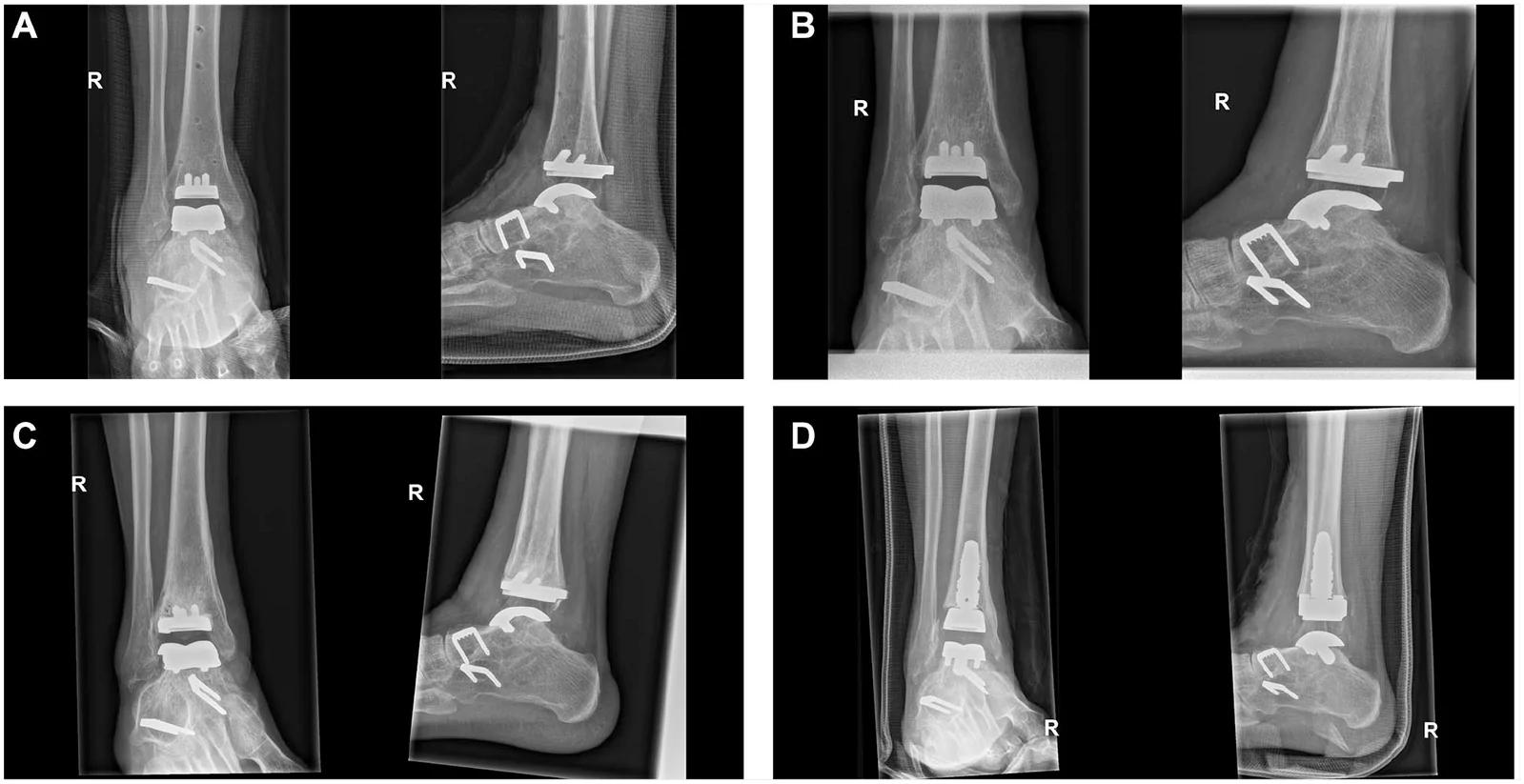
Revision due to aseptic loosening of the tibia component: (A) Postoperatively; (B) Loosening of tibia after 10 months; (C) Revision to cemented tibia and (D) Revision to Invision/Inbone implant.

One patient probably had a fracture of the medial malleolus during the early postoperative period. This was not diagnosed until the 3 months visit, where radiographically evident impaction of the tibial component was found. The arthroplasty was revised using an Inbone tibial component, and osteosynthesis of the medial malleolus (Figure 3).

**Figure 3.**
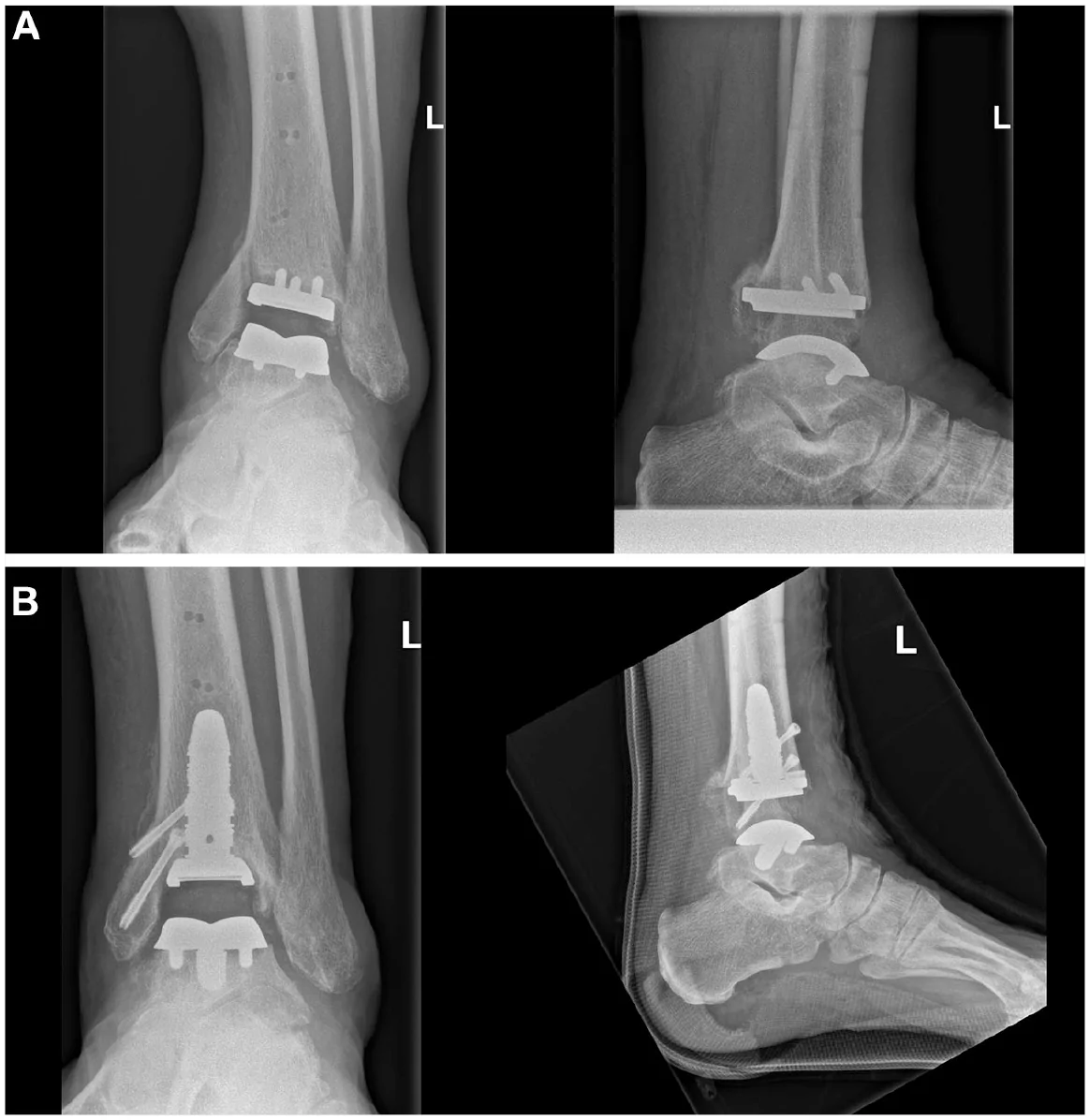
Revision due to fracture of the medial malleolus: (A) 3 months after surgery and (B) After revision to Inbone implant.

Three patients had reoperations not involving the primary TAA; 1 case of debridement of a superficial surgical site infection, 1 case of debridement and epidermal skin grafting of a superficial wound necrosis, and 1 osteosynthesis of a traumatic medial malleolus fracture obtained the first postoperative day.

#### Reoperations during the second year

One hundred patients had 2 years of follow-up time, and one of them was revised during the second year. This reoperation was due to malalignment of the hindfoot. This was partly corrected preoperatively with a calcaneal osteotomy prior to implantation of the TAA, but due to persistent malalignment, the patient was reoperated with a distal tibial osteotomy and the polyethylene insert was exchanged to a thicker one.

### Radiographic Outcomes

There were 95 radiographic examinations available 1 year after primary surgery. We found radiolucent lines in 5 (5.3%) patients, cysts in 3 (3.2%) patients, and both radiolucent lines and cysts in 1 (1%) patient. In total there were findings of radiolucent lines and/or cysts in 9 (9.5%) cases, 1 year after primary surgery. Two of these patients complained of pain in the ankle joint. In one of these patients, the pain spontaneously subsided over time. The other patient was examined with computer tomography, which showed no signs of loosening of the implant.

### Clinical Outcome Measures

One year postoperatively, all clinical outcome measures had improved in comparison to preoperative scores. There was an increase in mean AOFAS, EQ5D-3L, and EQ5D-VAS and a decrease in MOxFQ (inverse scale), all changes were statistically significant (Table 4).

**Table 4.** Clinical Outcome Measures Preoperatively and 1-Year Postoperatively.

Outcome measures	N	Preoperative mean (95% CI)	1-year postoperative mean (95% CI)	Mean score Δ (95% CI)	*P value*
**AOFAS**	71	45.2 (41.9 to 48.4)	80.9 (76.9 to 84.9)	35.9 (31.9 to 39.9)	<.01
**MOxFQ**	67	66.4 (63.5 to 69.4)	19.6 (14.6 to 24.5)	-46.9 (-41.7 to -52.1)	<.01
**EQ5D**	71	0.44 (0.39 to 0.48)	0.79 (0.75 to 0.85)	0.36 (0.30 to 0.41)	<.01
**EQ5D-VAS**	68	59.8 (55.2 to 64.5)	73.1 (68.4 to 77.7)	13.2 (7.8 to 18.7)	<.01

American Orthopaedic Foot and Ankle Society score (AOFAS) 0-100, Manchester Oxford foot and ankle scale (MOxFQ) 100-0, EuroQoL-5D (EQ5D) and EQ5D-quality of life (EQ5D-VAS) 0-100.

We did not find any statistically significant linear associations between ΔAOFAS (Figure 4 A-D) or ΔMOxFQ (Figure 4 E-H) and BMI, age, sex, or primary diagnosis.

**Figure 4.**
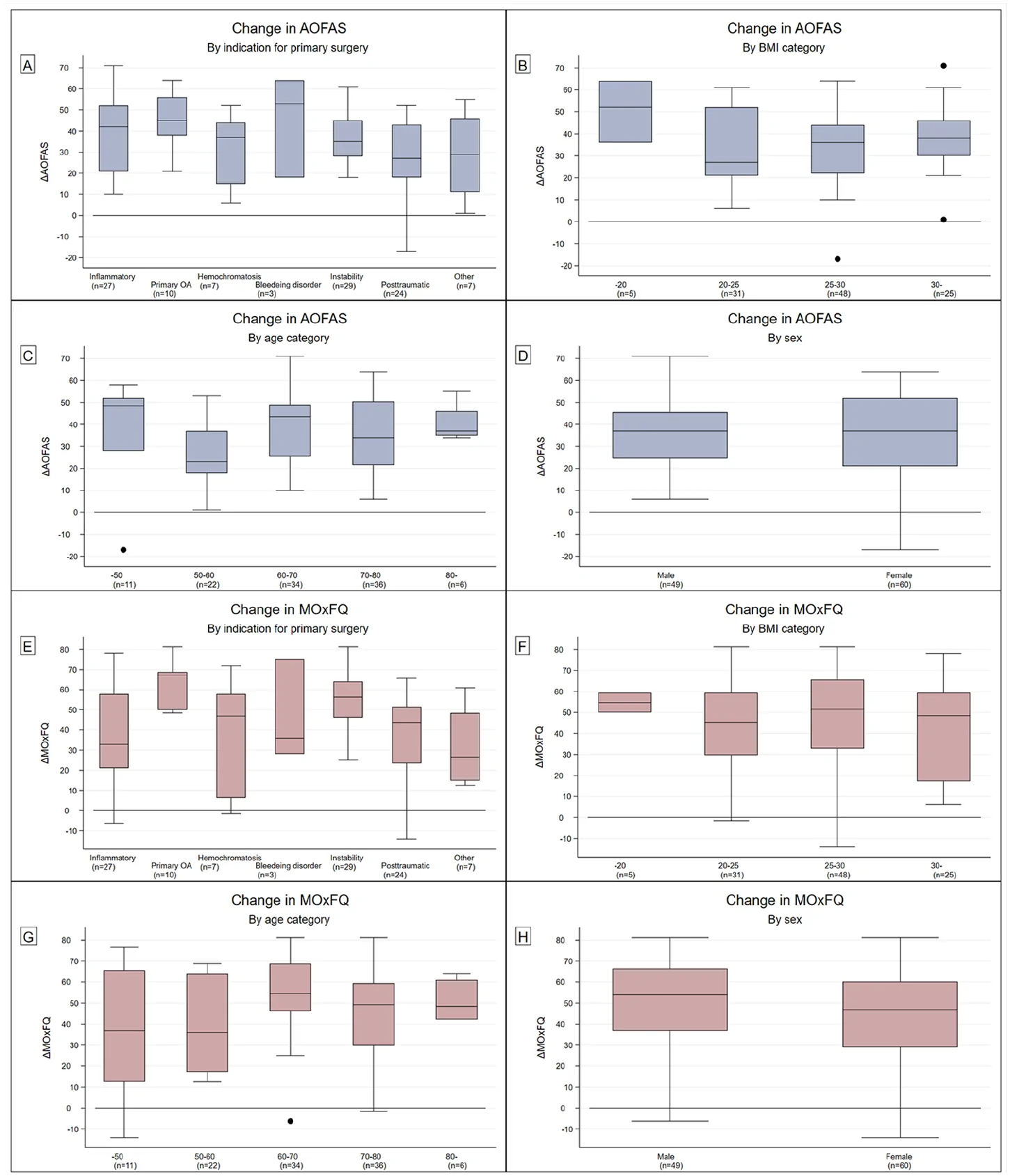
A-H: Outcome measures in association with patient factors.

## Discussion

We found the 1-year implant revision rate after primary TAA using the Infinity ankle system to be 2.8%, including 1 case of aseptic tibial loosening (0.9%). The overall rate of complications requiring secondary surgery was 6.6% after 1 year. Among the 94.3% of cases with at least 2 years follow-up at the cut-off date for these analyses, there was only 1 additional reoperation. Patients reported considerable improvement in physical function and quality of life. Our results show numerically lower revision rates compared to 3 earlier published studies on the Infinity system, which reported early revision rates at 3.1% to 4.7% due to aseptic loosening,^7,8,17^ and similar results to recent studies that report a survivorship of 94% to 98.2% after approximately 2 years.^11,12^

There was significant improvement in all outcome measures. The mean improvement reported for MOxFQ was 46.9 (95% CI: 41.7 to 52.1), which is well above the reported minimal detectable change (MDC) of 14.18.^18^ These findings are supported by the results for the standardized EQ-5D 3L measure of health-related quality of life, where the minimal clinical important difference (MCID) is reported to be 0.18,^19^ and among our patients showed an overall improvement of 0.36 (95% CI: 0.30 to 0.41).

Previous studies show similar outcome scores when comparing ankle replacement to ankle arthrodesis.^20,21^ In the only randomized controlled trial available, fixed bearing ankle arthroplasty was found to be superior to arthrodesis in a post-hoc subgroup analysis.^21^ Historically, a total ankle replacement has been reserved for selected patients, but with more knowledge and improved implants, this surgery may be considered for a larger proportion of patients. We believe these patients should be monitored, to determine implant survival over longer time periods. It would also be of interest to obtain knowledge of outcomes of revision from ankle prosthesis to either revision prosthesis or secondary arthrodesis.

We found presence of periprosthetic radiolucent lines and cysts in less than 10% of our cases 1 year after primary surgery. Rushing et al^12^ reported findings of radiolucent lines in 34.5% of cases 1 year postoperatively, Saito et al reported radiolucent lines in 31%, Penner et al^6^ described radiolucent lines only in patients re-operated due to aseptic loosening (2.9%), and a large multicenter study by Townshend et al. reported radiolucent lines in 14.2% of cases after 1 year.^7,10^ To further understand the clinical relevance of these findings, and whether they are an early sign of aseptic loosening, longer follow-up time and larger data sets are required.

The main limitation of this study was that some patients who experienced complications did not attend the regular 1-year visit, as they had shifted to another follow-up regimen; these cases were thus not included in the clinical outcome score analyses, and this leads to a survivorship bias. Also, some patients living in remote parts of the country were unable or unwilling to travel to attend the 1-year clinical visit, resulting in missing outcome data. The follow-up has been part of ordinary clinical practice, at predefined intervals at 3 months, 1 year, and 5 years. It is not part of our routine to distribute questionnaires to patients not attending controls, and the registry is not designed to obtain clinical outcome measures and radiographic findings after 2 years. The follow-up time was short, considering the expected survival time of the implant, and there will be a need for further observational studies with longer follow-up times.

The strengths of this study include outcome scores collected at predefined time-points. All patients operated with TAA at our hospital are included in the registry, without specific inclusion- or exclusion criteria, systematically recording a wide range of operative, clinical, patient-reported, and radiographic outcomes. Furthermore, there was conformity in the operative technique, postoperative care, and follow-up by the same group of surgeons and physiotherapists. The study results thus reflect the outcomes of TAA in a relatively large number of cases in a real clinical setting at a medium sized center with no commercial affiliations.

## Conclusion

Our results show an acceptable short term rate of aseptic loosening, revisions, complications, and radiolucent lines with the Infinity total ankle system. We report satisfactory improvements in AOFAS, MOxFQ, and EQ5D scores 1 year postoperatively. As most patients will live for decades after ankle replacement surgery, long-term follow-up is very important to evaluate this ankle implant, and this cohort will be followed further.
